# Saccular Aneurysm Models Featuring Growth and Rupture: A Systematic Review

**DOI:** 10.3390/brainsci10020101

**Published:** 2020-02-13

**Authors:** Serge Marbacher, Stefan Wanderer, Fabio Strange, Basil E. Grüter, Javier Fandino

**Affiliations:** 1 Department of Neurosurgery, Kantonsspital Aarau, Aarau 5000, Switzerlandbasil.grueter@ksa.ch (B.E.G.);; 2 Cerebrovascular Research Group, Department for BioMedical Research, University of Bern, Bern 3000, Switzerland

**Keywords:** animal model, growth, aneurysm rupture, saccular, intracranial aneurysm

## Abstract

Background. Most available large animal extracranial aneurysm models feature healthy non-degenerated aneurysm pouches with stable long-term follow-ups and extensive healing reactions after endovascular treatment. This review focuses on a small subgroup of extracranial aneurysm models that demonstrated growth and potential rupture during follow-up. Methods. The literature was searched in Medline/Pubmed to identify extracranial in vivo saccular aneurysm models featuring growth and rupture, using a predefined search strategy in accordance with the PRISMA guidelines. From eligible studies we extracted the following details: technique and location of aneurysm creation, aneurysm pouch characteristics, time for model creation, growth and rupture rate, time course, patency rate, histological findings, and associated morbidity and mortality. Results. A total of 20 articles were found to describe growth and/or rupture of an experimentally created extracranial saccular aneurysm during follow-up. Most frequent growth was reported in rats (*n* = 6), followed by rabbits (*n* = 4), dogs (*n* = 4), swine (*n* = 5), and sheep (*n* = 1). Except for two studies reporting growth and rupture within the abdominal cavity (abdominal aortic artery; *n* = 2) all other aneurysms were located at the neck of the animal. The largest growth rate, with an up to 10-fold size increase, was found in a rat abdominal aortic sidewall aneurysm model. Conclusions. Extracranial saccular aneurysm models with growth and rupture are rare. Degradation of the created aneurysmal outpouch seems to be a prerequisite to allow growth, which may ultimately lead to rupture. Since it has been shown that the aneurysm wall is important for healing after endovascular therapy, it is likely that models featuring growth and rupture will gain in interest for preclinical testing of novel endovascular therapies.

## 1. Introduction

Increased understanding of the complex pathobiology of intracranial aneurysm (IA) growth, rupture, and the effects of endovascular therapy depends on epidemiological data analysis, clinical findings, histopathology of IA samples obtained during surgery, and gene linkage analysis [1,2,3,4,5] Experimental work using animal models of IA are needed to delineate the biological mechanisms of IA formation and growth, and to establish new medical and endovascular therapies and materials to prevent IA rupture. Cerebral aneurysm models can be divided into two large groups: Intra- and extracranial models [6]. 

There is a growing body of evidence that the aneurysm wall condition influences the healing response and long-term durability after endovascular therapy [7,8,9,10]. Most available extracranial aneurysm models feature healthy non-degenerated aneurysm pouches with stable long-term follow-ups and extensive healing reactions after endovascular treatment [11]. This review focuses on a small subgroup of extracranial saccular aneurysm models that demonstrate growth and potential rupture during follow-up. It is likely that this subgroup of models will become more important for challenging the testing of devices prior to their clinical application [6,7,12]. This systematic review provides a comprehensive overview of available techniques and associated characteristics of extracranial aneurysm models featuring growth and rupture. Furthermore, this summary serves as reference for the development of novel models and supports researchers in the planning and execution of their future experiments. 

## 2. Materials and Methods

### 2.1. Literature Search

The literature was searched in Medline/Pubmed on November 31, 2017 to identify extracranial in vivo saccular aneurysm models featuring growth and rupture using a predefined search strategy. Briefly, we used the following key words: “murine”, “rat”, “rabbit”, “canine”, “primate”, “cat”, “pig”, ”sheep”, and “goat” in combination with “intracranial aneurysm” using the Boolean operator [AND]. The search was restricted to animals and two investigators (SM and FS) independently screened titles and abstracts for eligible studies and removed duplicates. Full text analysis of the remaining articles determined their final eligibility. Uncertainties by the two investigators were discussed with a third examiner (BG). Cross-references were searched until no further studies were identified. The search algorithm was in accordance with the PRISMA guidelines. 

### 2.2. Eligibility Criteria and Analyzed Features

We considered all preclinical extracranial saccular aneurysm models with documented growth and/or rupture. We excluded in vitro experiments, studies on intracranial vessels, studies published in a language other than English, articles designed for the study of thoracic or abdominal aortic aneurysms, and review articles. From each study included in the final analysis we recorded the following: authors, year of publication, aneurysm model category (sidewall, terminal, stump, bifurcation, and complex), species, detailed technique of aneurysm creation, aneurysm pouch characteristics (vital or modified, arterial or venous), initial size and location of the aneurysms, time for model creation, growth rate and time course of growth, size of increase (as percentage of baseline), rupture rate and time course, patency rate, mortality and morbidity rate, and histological findings.

## 3. Results

**Figure 1:**
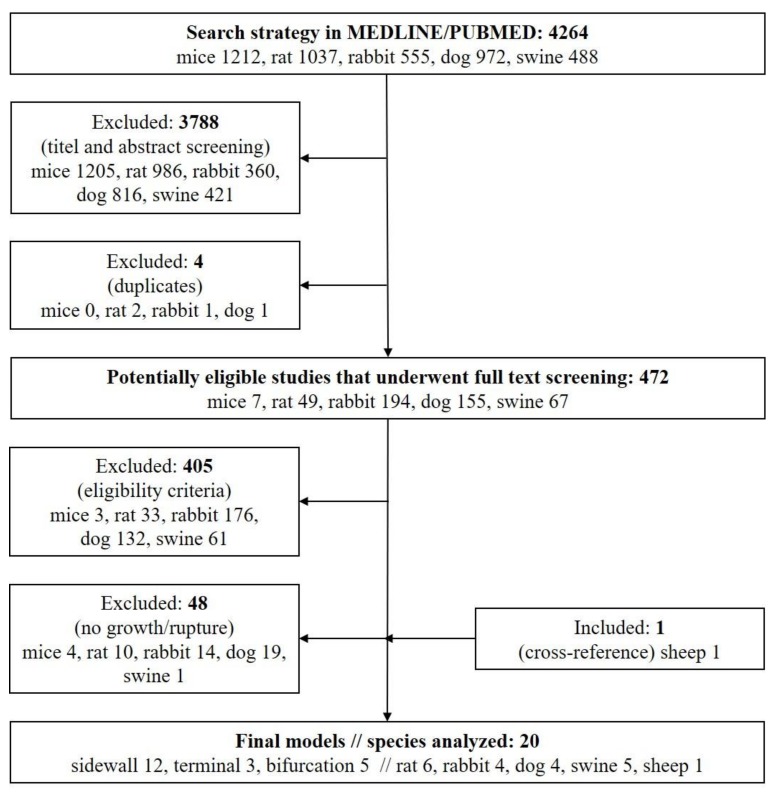
PRISMA flow chart for study selection.

A total of 20 articles were found that described growth and/or rupture of an experimentally created extracranial saccular aneurysm. The initial electronic search yielded 4264 potential studies. Of these, 3788 articles were excluded after title and abstract screening and 4 articles were excluded after identification of duplicates. The remaining 472 articles underwent full text analysis. Of those, 405 studies were excluded according to the predefined eligibility criteria. Another 48 studies describing various saccular aneurysm models were excluded because none of the reported techniques resulted in growth or rupture of the created aneurysms. One study was added by cross-referencing (Figure 1).

Growth and/or rupture of experimental aneurysms were found in three types of models: sidewall (*n* = 12) [3,8,13,14,15,16,17,18,19,20,21,22], bifurcation stump (*n* = 6) [14,23,24,25,26,27], and terminal (*n* = 3) [28,29,30]. Most frequent growth was reported in rats (*n* = 6) [8,14,16,23,24,25], followed by rabbits (*n* = 4) [13,21,26,29], dogs (*n* = 4) [19,27,28,30], swine (*n* = 5) [3,17,18,20,22], and sheep (*n* = 1) [15]. Except for two studies reporting growth and rupture within the abdominal cavity (abdominal aortic artery; *n* = 2) [8,16] all other aneurysms were located at the neck of the animal (common carotid artery; *n* = 18). The identified 20 models used in *n* = 14 venous pouches (in *n* = 2 of them inverted venous pouches [8,30]), in *n* = 3 modified arterial pouches (*n* = 2 porcine elastase [26,29] and *n* = 1 sodium dodecyl sulfate [8]), and in *n* = 3 direct mechanical arterial wall weakening [13,23,24] to create growing and rupture-prone aneurysms. Time for aneurysm creation was reported in only two studies (180 minutes each for a terminal model in dogs [28] and rabbits [29]).

Almost half (*n* = 9 out of 20) of the models demonstrated growth only without associated rupture during follow-up. The volume increase varied greatly between the models used and ranged between tiny blebs [23] and a 10-fold increase [8] in initial size. Most models (*n* = 17 out of 20) reported only modest increase, with stabilization at further follow-up. The largest increase in aneurysm volume was found in rat sidewall aneurysm models created in the abdominal cavity. Growth rate and time course of growth ranged from 23% to 100% and from weeks to months, respectively.

More than half (*n* = 11 out of 20) of all identified models reported rupture during follow-up. In three out of these eleven models the aneurysm wall was modified at the time of creation [8,26,29]. Intraluminal aneurysm thrombosis was present in 9 out of 11 models featuring rupture. Rupture occurred within a few days and up to months after aneurysm creation. Except for a single case of rupture within one day all other ruptures occurred later than day 3 after creation, irrespective of the model applied. The rate of rupture ranged from 7% to 100% depending on the model used. Associated morbidity and mortality ranged from 0% to 50%. Three studies did not report associated morbidity and mortality rate.

The predominant histological findings in growing and ruptured aneurysms are: unorganized intraluminal thrombus, incomplete neointima formation with partial aneurysm recurrence, marked inflammatory cells within unorganized thrombus and aneurysm wall, hemorrhagic transformation of the aneurysm wall with intramural loss of endothelial cells, smooth muscle cells, and degradation of extracellular matrix components. All details of each model and associated characteristics are summarized in Table 1.

## 4. Discussion

Most extracranial aneurysm models differ from human saccular aneurysms not only in their histology, but their reluctance towards growth and rupture. In consequence, aneurysm growth and/or rupture during follow-up are rare events. This review demonstrates that the following characteristics seem to be associated with growth and rupture of extracranial saccular aneurysms, regardless of the species or model used: intraluminal aneurysm thrombosis, intraluminal and intramural inflammation, endothelial and mural cell loss, and hemorrhagic transformation of the aneurysm wall. Most of the identified 20 extracranial saccular aneurysm models were of sidewall type and featured only short-term aneurysm maturation rather than true aneurysm growth during follow-up. 

After pioneering work on aneurysm creation by direct vessel manipulation on extra- and intracranial arteries by McCune et al. [31] and White et al. [32] it was Troupp and Rinne [13] who demonstrated the growth of sidewall carotid aneurysms in rabbits created by an arteriotomy glued with methl-2-cyanoacrylate. They found significant increase in size in 30% of aneurysms over a time of 1 to 5 months. Many models demonstrate maturation by means of aneurysm enlargement in the first weeks after creation but remaining stable thereafter [24,25,26,28,30]. Nishikawa et al. [14] and Gao et al. [24] demonstrated growth by means of maturation in rat venous pouch sidewall and bifurcation aneurysms. Fujiwara et al. [26] found a similar increase in size within the first four weeks with a further stable course of up to 4 months in an elastase arterial bifurcation stump model in rabbits. Naggara et al. [30] also found a maturation/growth within the first month and then stable course up to 10 months after creation of venous pouch terminal aneurysms in dogs. This may be explained by the absence of true perivascular inflammation and normal cellularity of the aneurysm walls. This healthy venous vascular tissue that the aneurysms were made of may have been able to organize to allow cell migration, and to synthesize a new extracellular matrix, eventually resulting in aneurysm healing. In contrast, the largest increase in size and true growth (ten-fold increase in size compared to baseline) was found in a rat abdominal aortic arterial pouch sidewall aneurysm model [8]. This remarkable growth was probably only possible due to aneurysm wall decellularization and the fact that the abdominal cavity is less restrictive than the subcutaneous soft tissue of the neck region.

More than half (55%) of the identified models demonstrated rupture of the experimental aneurysm during follow-up. In almost half of the reported models that demonstrate rupture, the aneurysm wall had been modified at the time of creation (Table 1). However, in all these models that featured rupture, the aneurysm wall was either weakened during creation (chemically or mechanically) or demonstrated marked wall degeneration (inflammation and intraluminal thrombosis) at autopsy. Stehbens [15] reported in 1979 that 30% (8/27) of venous sidewall aneurysms created at the common carotid artery in sheep ruptured within three weeks after creation. All these ruptured aneurysms contained macroscopic thrombus. Raymond et al. [3] demonstrated that 100% (7/7) of giant and 50% (2/4) of small-neck swine common carotid artery sidewall venous pouch aneurysms ruptured within 1–2 weeks after creation. They found that many areas of the aneurysm wall showed a lack of smooth muscle cells and elastic fibres but had inflammatory cells infiltrating the wall, along with hemorrhagic transformation of the media, adventitia, and perianeurysmal tissue. Yang et al. [29] presented a terminal rabbit aneurysm model with an arterial pouch modified with both elastase and collagenase. In this model, aneurysms grew within the first 1–2 weeks in 100% of cases (10/10) and 33% (3/9) of them ruptured within 4 weeks after creation. Histopathology revealed that the aneurysm wall was composed only of a thin layer of acellular fibrous tissue. Decellularization of the aneurysm wall in a sidewall rat aneurysm model resulted in aneurysm growth in 33% (4/12) and rupture in 25% (3/12) [8]. Decellularized aneurysms in this model demonstrated inflammation and damage to the aneurysm wall and marked neutrophil accumulation in the luminal thrombus.

In summary, loss of mural cells and chronic aneurysm wall inflammation is a crucial factor for both saccular aneurysm growth and rupture. It has been demonstrated that aneurysms that lost mural cells also lost their ability to organize luminal thrombus and to form a neointima [8,33]. Instead, ongoing inflammation results in destructive wall remodeling, further mural cell loss and thinning of the vascular wall which in turn favors further aneurysm growth and rupture. Thus, in order to establish a model which can reflect true aneurysm growth and rupture instead of just a short-term maturation, artificial rarefication of mural cells is necessary.

In addition to intracranial animal models for the study of aneurysm formation and rupture, it will be essential to further develop larger extracranial animal models that will allow to study embolization devices and healing processes in growing and rupture-prone aneurysms. Although most valuable, aneurysm models featuring growth and rupture are ethically questionable due to potential sudden death. Close monitoring (e.g., ultrasound imaging) to regularly check for the hemodynamic situation is recommended in all experimental aneurysm models featuring growth and rupture [3,8,34]. 

## 5. Conclusions

Extracranial saccular aneurysm models with growth and rupture are rare. Most of these models presented the increases in aneurysm size by means of maturation rather than ongoing degradation of the aneurysm wall and true growth that ultimately results in aneurysm rupture. Histological findings suggest that degradation of the wall (either by direct manipulation at the time of creation or indirect weakening mediated through intraluminal thrombosis and inflammation) is essential for rupture of an artificially created saccular aneurysm model. Since it has been shown that the aneurysm wall is important for healing after endovascular therapy, it is likely that models featuring growth and rupture will gain interest in the preclinical testing of novel endovascular therapies. 

## Figures and Tables

**Table 1:** Detailed characteristics of aneurysm models featuring growth and rupture.

#	Author (Year)	Animal	Location // Size (Baseline)	Model (Pouch) // Time for Creation	Modified Wall // Thrombus	Growth Rate and Time Course // Patency Rate // Size Increase from Baseline (%)	Rupture Rate and Time Course // Mortality and Morbidity	Histological Findings
1	Troupp and Rinne (1964) [13]	Rabbit	Rt CCA // NR	Sidewall // NR	Yes (arteriotomy glued with Methyl-2-Cyanoacrylate) // NR	32% (16/50) within 4–21 weeks // 38% (6/16) within 4–13 weeks // NR	None // 6% (3/50) mortality	NR
2	Nishikawa et al. (1976) [14]	Rat	CCA // 2.15 ± 0.39 mm (length) × 1.55 ± 0.34 mm (width) × 0.88 ± 0.36 mm (height)	Sidewall and true bifurcation (venous pouch, AFV) // NR	No // 4% (4/112)	Growth within the first week // 96% (108/112) // 24% (length), 25% (width) and 42% (height)	8% (9/112) rupture in both models (sidewall and true bifurcation) // 4.46% (5/112)	Thickening of the aneurysm wall, when the aneurysm had existed for a long time
3	Stehbens (1979) [15]	Sheep	CCA // NR	Sidewall (venous pouch, EJV) // NR	No // 41% (11/27)	No notable growth // NR // NR	30% (8/27) within 3 weeks // 30% (8/27) within 3 weeks	Detailed description of histological changes in the aneurysm sac and parent artery. All ruptured aneurysms contained macroscopic thrombus
4	Young et al. (1987) [23]	Rat	CCA // 2 × 2 mm	True bifurcation // NR	Yes (external mural excision) // NR	Aneurysms grew into tiny blebs of various shape and sizes at 3–12 weeks FU // NR // NR	55.5% (5/9) // NR	Aneurysms were usually small and broad-based with noticeably thin walls
5	Gao et al. (1990) [24]	Rat	CCA // 0.8 ± 0.3 mm (length) × 0.7 ± 0.2 mm (width) ± 0.4 ± 0.1 mm (height)	True bifurcation // NR	Yes (transluminal removal of the tunica intima and media) // 0% (0/20)	Significant growth of all 20/20 aneurysm within the first 2 months remained stable until 3 months FU // 70% (14/20) after 2 months, 60% (6/10) after 3 month // 37.5% length, 28.57% width, 50% height	0% (0/20) // 0% (0/20)	No thrombosis, endothelial cells covered smooth surface. IEL and tunica media absent; regenerative elastic fibers without pattern and dispersive. Disorderly arranged fibroblast-like between the collagenous and elastic fibers. Vasa vasorum and few foam cells occasionally in the experimental wall tunica adventitia intact and infiltrated by some mononuclear cells and foreign body giant cells
6	Sadasivan et al. (1990) [16]	Rat	AA // 3 mm	Sidewall (venous pouch, IJV) // NR	No // 6.45% (4/62)	Growth occurred after wrapping with cotton or polyvinyl alcohol // 100% (62/62) // NR	NR // NR	All giant aneurysms (*n* = 4) were partially thrombosed. Two in each wrapping group
7	Graves et al. (1993) [28]	Dog	Both CCA // 15 mm (width), 21 mm (height)	Terminal (venous pouch, EJV) // 180 minutes	No // NR	Increase in size over time at 13 weeks (9–17 weeks) // 100% (6/6) // average increase 33% width, 9.52% height	0% (0/6) // 0% (0/6)	NR
8	Byrne et al. (1994) [17]	Swine	CCA // 15-20 mm (length)	Sidewall (venous pouch, EJV) embolized with GDC // NR	No // 14.28% (1/7)	Tendency for growth in aneurysms with partial thrombosis // 14.28 (1/7) after 2–3 weeks // NR	100% (4/4) of untreated aneurysm within 4 ± 0.5 days; 75% (3/4) of partial (<90%) occlusion using GDC within 4 ± 1 days // 50% (7/14)	Marked edema and acute inflammatory infiltration of the whole wall, wall dissection, and necrosis of smooth muscle fibers
9	Kirse et al. (1996) [25]	Rat	Both CCA // 1.40 mm (width) × 3.125 mm (height)	Artificial bifurcation (venous pouch, EJV) // NR	No // 33.33% (4/12)	1.45 mm (width), 3.45 mm (height) after 1 week, 2.4 mm (width), 3.875 mm (height) after 3 weeks, 2.1 mm (width), 4.175 mm (height) after 3 months // 100% (12/12) // Average volume increases 21.5% after 1 week, 96% after 3 weeks and 145% within 3 months	NR // NR	Small adventitial collections of lymphocytes, some pigment-laden macrophages, and focal foreign body giant cell reaction to suture material. The endothelial surfaces were intact and continuous and the lumens patent
10	Raymond et al. (1999) [18]	Swine	CCA // NR	Sidewall (venous pouch, EJV) embolized with collagen sponges 95% (25/30) or Guglielmi Detachable coils 5% (5/30) // NR	No // NR	NR // 100% (25/25) // NR	80% (4/5) rupture of residual aneurysm after embolization within 3–5 days // 16% (5/30) mortality	Healing responses following embolization of porcine aneurysms with GDC or Gelfoam sponges were essentially similar at 3 weeks
11	Fujiwara et al. (2001) [26]	Rabbit	CCA // NR	Bifurcation stump // NR	Yes (arterial pouch, CCA modified with porcine elastase (Sigma, St. Louis) for 20 minutes in 66.66% (6/9)) // NR	100% growth rate (6/6) within 1 month (day 3 3.2 ± 0.6 mm (width), 6.0 ± 1.3 mm (height); day 14 4.1 ± 1.7 mm (width), 8.3 ± 1.9 mm (height); 35 days 5.0 ± 0.9 mm (width), 10.0 ± 2.2 mm (height) with stable course up to 4 months in the elastase group // 100% (9/9) // NR	0% (0/9) // 0% (0/9)	NR (control animals without elastase infusion did not show dilation of the stump at any timepoint (3–21 days) after aneurysm creation)
12	Yang et al. (2001) [19]	Dog	CCA // 6–8 mm (diameter), 3–4 mm (neck)	Sidewall (venous pouch, EJV) embolized with CAP // NR	No // 84.61% (11/13) with CAP treatment	16.66% (1/6) of partially thrombosed aneurysm enlarged between 4–8 weeks // 25% (3/12) // NR	33.33% (2/6) of total and subtotal occluded aneurysms ruptured at day 4 and 5 // 33.33% (2/6)	Endothelial cells and basal membrane were destroyed. Fibrous cells and SMC showed obvious degeneration. Inflammatory cells most prominent 1–2 weeks after thrombosis
13	Murayama et al. (2003) [22]	Swine	CCA // 8–12 mm (diameter), 7 mm (neck)	Sidewall (venous pouch, EJV) embolized with GDC or Matrix // NR	No // GDC 100% (23/23) and 100% (26/26) after 6 months	NR // NR // 14.6% from baseline to day 14 in the GDC group, 19.68% in the Matrix group; 4.09% from baseline for the GDC group after 3 months, 6 months NA for the GDC- and Matrix group	23% (3/13): 5 days (2/13) and 12 days (1/13) after GDC embolization // 11.5% (3/26)	Unorganized intraluminal clot (5 day) and large neck hematoma (day 12), rupture point at the dome of the venous pouch
14	Becker et al. (2007) [20]	Swine	CCA // 8.9 mm (height), 8.2 mm (width), 7.7 mm (depth)	Sidewall (venous pouch, EJV) embolized with calcium alginate // NR	No // 100% (8/8)	NR // 0% (0/8) in treatment group within 3 months, 100% (2/2) in control group of partial occlusion (<50%) within 8 days // NR	100% (2/2) of partial occlusion (<50%) after 6 and 8 days // 20% (2/10)	Inflammatory cell infiltration in aneurysm sac and neutrophil infiltration within unorganized thrombus
15	Yang et al. (2007) [29]	Rabbit	Both CCA // 8 mm (length)	Terminal // 180 minutes	Yes (arterial pouch, CCA modified with Hanks solution containing elastase (60 U/ml) for 20 minutes and collagenase typ I for 15 minutes) // 33.33% (3/9)	100% (9/9) within 1-2 weeks // NR // mean diameter increased 60% after 2 weeks (from 2.0 ± 0.1 mm to 3.2 ± 0.3 mm)	33.33% (3/9), one each after 1 day, 2 weeks, and 4 weeks // 40% (4/10)	Differentiation of tunica intima, media and adventitia was lost. Fragmentation of elastic laminar. Thinning of the wall composed of a thin layer of acellular fibrous tissue/collagen
16	Tsumoto et al. (2008) [27]	Dog	Both CCA // NR	Artificial bifurcation (venous pouch, EJV) // NR	No // 20% (1/5)	100% (5/5) within 10 months FU // 80% (4/5) // Significant increase after 10 months 18.7 ± 1.3 mm (height), 11.1 ± 1.9 mm (width), 8.1 ± 1.4 mm (neck))	0% (0/5) // 0% (0/5)	Aneurysms increase in size (height, width, and neck diameter) during the 1–4 months over a 10-month period. No significant differences in dimensions between 7 and 10 months
17	Naggara et al. (2010) [30]	Dog	Both CCA and IT // 13.9 ± 3.3 mm (fundus), 3.6 ± 1.2 mm (neck)	Terminal // NR	Yes (venous pouch, EJV, inverted) // NR	100% (16/16) within 1 month, then remained stable up to 10 months // 100% (16/16) at 9.0 ± 3.6 months FU // 19.19% fundus increase after up to 10 months (from 13.9 to 17.2 mm), 26.54% neck increase after up to 10 months (from 3.6 to 4.9 mm)	0% (0/16) // 0% (0/16)	NR
18	Ding et al. (2012) [21]	Rabbit	CCA // 2.4 ± 0.4 mm (neck), 4.3 ± 1.2 mm (width), 4.3 ± 1.4 mm (height)	Sidewall (venous pouch, EJV) // NR	No // NR	95% (38/40) increase within 3 weeks // 95% (38/40) aneurysms remained patent // 150% increase after 3 weeks (from 51 mm^3^ to 127.5 mm^3^)	0% (0/40) // 0% (0/40)	However, no data whether further growth occurred later than 1 month after creation
19	Raymond et al. (2012) [3]	Swine	Both CCA // group 1: 11.3 ± 2.6 mm (long axis), 6.7 ± 2.1 mm (short axis), 5.8 ± 0.6 mm (neck); group 2: 16.9 mm ± 4.0 mm (long axis), 8.1 mm ± 1.3 mm (short axis), 4.8 mm ± 1.1 (neck); group 3 26.1 ± 10.09 mm (long axis), 9.4 ± 1.4 mm (short axis), 5.8 ± 1.0 mm (neck)	Sidewall // NR	Yes (venous pouch, EJV, removal of endothelial lining) // 83.33% (20/24)	NR // 54.16% (26/48): group 1 remained patent at 2 weeks, partially occluded at 3 weeks, completely occluded in 4 weeks (*n* = 12); group 2 fully occluded at 2 weeks in 2 animals without rupture (*n* = 8); group 3 lesions clipped were confirmed to be completely occluded immediately postoperatively and at 7 days (n=6) // NR	50% (2/4) of small size with small neck in group 2 within 2 weeks, 100% (7/7) of giant size with wide neck in group 3 (untreated) within 1 weeks, 16.66% (1/6) of giant size with wide neck in group 3 (clipped); in total 41.66% (10/24) // 20.83% (5/24)	Intraluminal unorganized thrombus in allruptured aneurysm, many areas with loss of SMC and elastic fibers, inflammatory cells infiltrating the venous wall, hemorrhagic wall transformation
20	Marbacher et al. (2014) [8]	Rat	AA // 2.5 ± 0.3 mm (width) control group, 2.6 ± 0.2 (width) SDS group; 4.2 ± 0.4 mm (length) control group, 4.1 ± 0.6 mm (length) control group	Sidewall // NR	Yes (arterial pouch, syngeneic TA modified with SDS 0.1% for 6 hours to decellularize the wall) // 13% (3/10) in the control group after 4 weeks, 33% (2/6) in the SDS group after 4 weeks	33% (4/12) within 1 week, largest growth (43 × 38 × 24 mm) with 10x increase in size // 38% (3/8) in the control group after 4 weeks, 50% (3/6) in the SDS group after 4 weeks // up to 1000%	75% (3/4) earliest rupture within eleven days after creation // 18.75% (3/16)	Unorganized intraluminal thrombus, strong adventitial and wall inflammation, marked inflammatory cells in medial matrix, luminal thrombus with neutrophils. Wall dissection and mural hematomas. Loss of EC and SMC

Rt = Right; Lt = Left; NR = not reported; CCA = common carotid artery; AFV = anterior facial vein; EJV = external jugular vein; IJV = internal jugular vein; FU = follow-up; IEL = internal elastic lamina; AA = abdominal aorta; IJV = internal jugular vein; GDC = Guglielmi detachable coil; CAP = cellulose acetate polymer; SMC = smooth muscle cell; EC = endothelial cell; IT = innominate trunk; TA = thoracic aorta; SDS = sodium dodecyl sulfate.
